# Frequent activation of EGFR in advanced chordomas

**DOI:** 10.1186/2045-3329-1-4

**Published:** 2011-07-25

**Authors:** Barbara Dewaele, Francesca Maggiani, Giuseppe Floris, Michèle Ampe, Vanessa Vanspauwen, Agnieszka Wozniak, Maria Debiec-Rychter, Raf Sciot

**Affiliations:** 1 Department of Human Genetics, Catholic University of Leuven, University Hospitals, Leuven, Belgium; 2 Department of Pathology, Catholic University of Leuven, University Hospitals, Leuven, Belgium; 3 Laboratory of Experimental Oncology, Department of General Medical Oncology, Catholic University of Leuven, University Hospitals, Leuven, Belgium; 4 I-BioStat, Catholic University of Leuven, Leuven, Belgium, and Hasselt University, Hasselt, Belgium

## Abstract

**Background:**

Chordomas are rare neoplasms, arising from notochordal remnants in the midline skeletal axis, for which the current treatment is limited to surgery and radiotherapy. Recent reports suggest that receptor tyrosine kinases (RTK) might be essential for the survival or proliferation of chordoma cells, providing a rationale for RTK targeted therapy. Nevertheless, the reported data are conflicting, most likely due to the assorted tumor specimens used for the studies and the heterogeneous methodological approaches. In the present study, we performed a comprehensive characterization of this rare entity using a wide range of assays in search for relevant therapeutic targets.

**Methods:**

Histopathological features of 42 chordoma specimens, 21 primary and 21 advanced, were assessed by immunohistochemistry and fluorescent *in situ *hybridization (FISH) using *PDGFRB, CSF1R*, and *EGFR *probes. Twenty-two of these cases, for which frozen material was available (nine primary and 13 advanced tumors), were selectively analyzed using the whole-genome 4.3 K TK-CGH-array, phospho-kinase antibody array or Western immunoblotting. The study was supplemented by direct sequencing of *KIT, PDGFRB, CSF1R *and *EGFR*.

**Results:**

We demonstrated that EGFR is frequently and the most significantly activated RTK in chordomas. Furthermore, concurrent to EGFR activation, the tumors commonly reveal co-activation of alternative RTK. The consistent activation of AKT, the frequent loss of the tumor suppressor *PTEN *allele, the recurrent activation of upstream RTK and of downstream effectors like p70S6K and mTOR, all indicate the PI3K/AKT pathway as an important mediator of transformation in chordomas.

**Conclusions:**

Given the complexity of the signaling in chordomas, combined treatment regimens targeting multiple RTK and downstream effectors are likely to be the most effective in these tumors. Personalized therapy with careful selection of the patients, based on the molecular profile of the specific tumor, is anticipated.

## Background

Chordomas are rare tumors. With an incidence of about 0.05/100000/year, they account for less than 5% of all primary malignant bone tumors. Mainly adults between 40 and 60 years are affected, but cases of children presenting with chordoma were also rarely reported (5% of cases). These bone tumors arise from remnants of the fetal notochord, and hence occur along the midline, and most often in the caudal spine or the base of the skull. They are slowly growing masses with the tendency to destroy the surrounding bone and to infiltrate adjacent soft tissue. Initial symptoms usually relate to local progression of the disease. Chordomas infrequently metastasize to lung, bone, soft tissue, lymph nodes and skin. On histology at low power magnification they show prominent lobules separated by fibrous septa. The tumors may be arranged in chords or sheets or may be floating singularly in the abundant myxoid matrix often present. The current treatment for chordoma is predominantly surgery, followed by radiotherapy. Safe margins are often difficult to obtain because of the anatomical location of the tumors [1]. Unfortunately, standard chemotherapy was shown to be basically unsuccessful, which causes serious problems for managing patients with locally recurrent or metastatic disease. Survival rates of 5 and 10 years are 68% and 40%, respectively [2].

Cytogenetic studies in chordomas have revealed in general nearly diploid or rather hypodiploid karyotypes, with a number of numerical and structural rearrangements. Recurrent genetic events reported in chordoma include frequent losses of large parts of chromosomes 3, 4, 10 and 13 and the most commonly lost regions are 1p31-pter, 3p21-pter, 3q21-qter, 9p24-pter and 17q11-qter [3]. The most common gains affect the chromosome 5q and the entire chromosomes 7 and 20 [4,5]. Loss of heterozygosity at 1p36 was also found in familial chordomas, further supporting the hypothesis that an important tumor suppressor might be located at the distal part of 1p [6]. Importantly, the *CDKN2A *tumor suppressor gene, which maps to 9p21.3, is reported to be lost in a high percentage (60%) of chordomas [7,8]. In addition, loss of one copy of the *PTEN *tumor suppressor gene (located on 10q23.31) was found in 37% (7/19) of lesions, although no difference in PTEN expression level was shown by Western blotting [8].

In the literature, several RTK, specifically PDGFRA, PDGFRB, KIT, EGFR, MET and HER2, were reported to be expressed in chordoma by immunohistochemistry [9-12]. Given that RTK could prove to be essential for the survival or proliferation of chordoma tumor cells, targeting these RTK using antibodies or small molecule tyrosine kinase inhibitors (TKI) might offer new treatment options for chordoma patients. Interestingly, imatinib was found to have antitumor activity in patients with chordoma [13]. It was suggested that PDGFRB signaling might be implicated in the tumor growth, as imatinib-responding tumors were found to be immunohistochemically positive for PDGFRB. Expression of basic fibroblast growth factor (bFGF), transforming growth factor alpha (TGF alpha) and fibronectin was reported to correlate with an increased incidence of disease recurrence in chordoma [14]. Moreover, clinical response to imatinib in one case was accompanied by the inhibition of PDGFRB as demonstrated by Western blot [13]. In recent reports, Tamborini and co-workers characterized 22 chordomas by immunoprecipitation and antibody arrays. The activation of PDGFRA, PDGFRB, KIT, FLT3, CSF1R, EGFR, HER2, HER4, AXL and DTK was reported in these studies [8,11]. Notably, PDGFRB activation was found in 95% (21/22) of cases. The EGFR activation, mainly through EGFR/HER2 heterodimer formation, was also suggested. Other groups found EGFR activation in three out of three and in about 50% of chordomas evaluated by RTK antibody arrays and immunohistochemistry respectively [15,16]. Partial response of metastatic chordoma to combined cetuximab/gefitinib treatment suggests that EGFR targeted treatment may benefit chordoma patients [9]. In addition, expression of the MET oncogene has been reported in chordoma [10]. Of note, the MET oncogene is known to be expressed in various chondroid neoplasms, normal articular cartilage and fetal notochord [17,18]. Given their possible relationship to notochordal development and chondroid differentiation, further investigation is warranted to clarify the roles of these and other RTK in chordomagenesis.

Activities of effectors more downstream in the main RTK pathways were also recently described. The ERK1/2, AKT and STAT3 activity was demonstrated in 18 (86%), 16 (76%) and 14 (67%) of cases, respectively, by immunohistochemistry performed on 21 chordomas [15]. Furthermore, analysis of 22 chordomas by Tamborini and co-workers showed consistent ERK1/2 activation in all the cases, and activation of AKT in 20 (91%), mTOR in 18 (82%), and S6 in 16 (73%) of the tumors [8].

In the present study, we have performed a comprehensive molecular and biochemical analysis of 42 chordomas, focusing on the role of RTK and their downstream signaling pathway in chordoma development, in primary tumors or their recurrent/metastatic counterparts.

## Methods

### Patients and histopathology

The present study included 31 patients [16 women and 15 men; age range 18-84 (median 58 years)] (Table 1). In total, 42 tumor specimens from these patients were retrieved, of which 21 were annotated as primary tumors and 21 as recurrences or metastases (in the text further referred to as advanced cases). The primary chordomas originated from the spine (n = 9), the sacrum (n = 10), the clivus (n = 1), and the cervix (n = 1). Samples 10a and 10b represent primary samples from the same patient obtained by needle biopsy and subsequent surgical resection, respectively. Histopathological examination was performed on formalin fixed, paraffin embedded tissue. Five μm sections were used for routine hematoxylin and eosin (H&E) staining, and immunohistochemical staining was performed by the avidin-biotin-peroxidase complex method, using the following monoclonal (mc) and polyclonal (pc) antibodies: Pankeratin (mc, dilution 1:200; Serotec, Oxford, UK), Epithelial Membrane Antigen (EMA) (mc, 1:50; DAKO, Glostrup, Denmark), Multikeratin (mc, dilution 1:10; Novocastra, Newcastle Upon Tyne, UK), S-100 protein (pc, 1:300; DAKO) and Vimentin (mc, dilution 1:500, DAKO). In addition, the EGFR (EGFR PharmDxTM, DAKO) and HER2/ERBB2 (HercepTestTM, DAKO) staining kits were used. EGFR and HER2 protein expression was reported as membranous brown staining of neoplastic cells using a three-tier system ranging from "1+" to "3+".

**Table 1:** Pathologic description of chordoma cases and results summary

Cases	Gender	Age	Tumor status	Site	Immuno		FISH				Proteome profiler array		TK Mut	Western		TK aCGH
								
					EGFR	HER2	EGFR	HER2	PDGFRB/CSF1R	PTEN	P-EGFR	P-PDGFRB		EGFR	PDGFRB	
1a	F	56	P	Spinal	1+	neg	dis.	dis.	polys.	nd	nd	nd	nd	nd	nd	nd
1b			R	Spinal	neg	nd	dis.	dis.	polys.	nd	weak	weak	nd	nd	nd	nd
2a	M	33	R	Spinal	1+	nd	dis.	dis.	polys.	nd	nd	nd	nd	nd	nd	nd
2b			M		3+	nd	dis.	dis.	polys.	nd	nd	nd	neg	E	E	nd
3	F	43	R	Spinal	3+	neg	polys.	dis.	dis.	dis.	strong	weak	neg	nd	nd	table 2
4a	M	62	P	Spinal	neg	nd	dis.	dis.	dis.	dis.	nd	nd	nd	nd	nd	nd
4b			M		2+	nd	polys.	dis.	polys.	monos.	interm	weak	neg	E	neg	table 2
5	F	75	R	Sacrum	3+	neg	polys.	polys.	polys.	polys.	nd	nd	neg	E/P	E	table 2
6	M	60	R	Clivus	3+	nd	dis.	monos.	dis.	dis.	strong	interm.	nd	nd	nd	nd
7a	F	62	P	Sacrum	neg	nd	polys.	dis.	dis.	dis.	nd	nd	nd	nd	nd	nd
7b			R	Sacrum	2+	nd	polys.	monos.	dis.	monos.	nd	nd	neg	E/P	E	table 2
8	M	36	P	Clivus	neg	nd	dis.	dis.	dis.	nd	nd	nd	nd	nd	nd	nd
9	M	52	R	Coccyx	1+	1+	monos.	dis.	dis.	dis.	nd	nd	neg	E/P	E	table 2
10a	F	41	P	Spinal	neg	nd	polys.	dis.	loss	dis.	nd	nd	nd	nd	nd	nd
10b			P	Spinal	neg	nd	polys.	dis.	loss	monos.	nd	nd	neg	neg	E/P	table 2
10c			R	Spinal	neg	nd	polys.	dis.	polys.	nd	nd	nd	nd	nd	nd	nd
11	F	54	P	Cervical	2+	nd	polys.	dis.	dis.	nd	strong	weak	nd	nd	nd	nd
12a	M	55	P	Sacrum	3+	neg	l.l.amp.	dis.	dis.	monos.	nd	nd	neg	nd	nd	nd
12b			M		3+	2+	h.l.amp.	dis.	polys.	nd	strong	weak	nd	E/P	E	nd
13	M	80	R	Coccyx	2+	nd	polys.	polys.	polys.	polys.	interm	weak	neg	nd	nd	table 2
14	F	60	R	Sacrum	1+	nd	dis.	polys.	dis.	monos.	nd	nd	neg	E/P	E	table 2
15a	F	73	P	Spinal	neg	nd	dis.	dis.	dis.	monos.	nd	nd	neg	nd	nd	table 2
15b			R	Spinal	neg	nd	polys.	polys.	polys.	nd	nd	nd	nd	E	E	nd
16	M	84	R	Sacrum	1+	neg	dis.	dis.	dis.	dis.	nd	nd	neg	nd	nd	table 2
17a	F	58	P	Sacrum	2+	1+	polys.	dis.	dis.	nd	strong	interm.	neg	nd	nd	table 2
17b			R	Sacrum	3+	neg	polys.	dis.	dis.	nd	nd	nd	nd	nd	nd	nd
18	F	57	P	Sacrum	3+	neg	polys.	dis.	dis.	dis.	strong	weak	nd	nd	nd	nd
19	M	84	P	Lumbal	1+	nd	dis.	monos.	dis.	nd	nd	nd	nd	nd	nd	nd
20	M	81	P	Sacrum	3+	neg	l.l.amp.	dis.	polys.	nd	strong	weak	nd	nd	nd	nd
21	F	67	P	Sacrum	1+	1+	h.l.amp.	dis.	dis.	nd	interm	weak	nd	nd	nd	nd
22	F	47	P	Sacrum	1+	nd	dis.	dis.	dis.	nd	weak	weak	nd	nd	nd	nd
23	M	48	P	Spinal	nd	nd	dis.	dis.	dis.	nd	nd	nd	nd	nd	nd	nd
24	F	60	R	Clivus/nc	3+	nd	polys.	polys.	polys.	polys.	nd	nd	nd	nd	nd	nd
25	F	60	R	Sacrum	neg	nd	monos.	dis.	nd	nd	nd	nd	nd	nd	nd	nd
26	M	80	M		nd	nd	polys.	polys.	polys.	loss	nd	nd	nd	nd	nd	nd
27	M	48	P	Sacrum	1+	nd	dis.	dis.	nd	nd	nd	nd	nd	nd	nd	nd
28	F	18	P	Spinal	nd	nd	dis.	dis.	nd	nd	nd	nd	nd	nd	nd	nd
29	M	37	P	Spinal	3+	neg	dis.	dis.	nd	nd	nd	nd	nd	nd	nd	nd
30a	M	42	P	Coccyx	neg	neg	monos.	dis.	nd	nd	nd	nd	nd	nd	nd	nd
30b		58	R	Sacrum	2+	neg	monos.	dis.	nd	nd	nd	nd	nd	nd	nd	nd
31a	F	60	P	Sacrum	neg	1+	polys.	dis.	nd	nd	nd	nd	nd	nd	nd	nd
31b			R	Ilium	neg	nd	polys.	dis.	nd	nd	nd	nd	nd	nd	nd	nd

TK Mut., Tyrosine Kinase Mutations; P, primary; R, recurrence; M, metastasis; nc, nasal cavity; neg, negative; l.l.amp., low level amplification; h.l.amp., high level amplification;

polys., polysomy; dis., disomy; monos., monosomy; nd, not done; interm, intermediate; E, expressed; P, phosphorylated.

### Array-CGH (aCGH) analysis

Array-CGH experiments were performed as previously described on DNA extracted from 11 tumors (Table 2) [19]. For genomic profiling that included the evaluation of all 90 TK known in humans, the 4.3 K genomic DNA tyrosine kinase array (TK-aCGH) was manufactured at the Microarray Facility of the Flanders Interuniversity Institute for Biotechnology, KULeuven [20]. In short, the Sanger 1 Mb Clone Set containing 3527 BAC/PAC clones was supplemented with 800 clones from 32 K CHORI BAC/PAC library, which specifically covers all known human TK, and these two clone sets were spotted together in duplicate on Code Linked Slides (AP Biotech, US). The complete list of these clones is available upon request. The array-CGH data were statistically analyzed with aCGH-smooth, software especially designed for the analysis of heterogeneous samples [21].

**Table 2:** Gains and losses in chordoma using whole-genome 4.3 K TK-CGH-array

	Case 3	Case 4b	Case 5	Case 7b	Case 9	Case 10b	Case 13	Case 14	Case 15a	Case 16	Case 17a
**Gains**	1q11-qter 7 8q11.21-qter 10pter-p11 20	5 7	2pter-p12	16q12.2-q22.1	n.d.	7	13q31.2-qter	1q11-qter 2 12pter-q24.23 17q12.1-qter X	n.d.	n.d.	n.d.

**Losses**	3pter-p11.1 8pter-p12 9 14 16q23.2-q24.3	1pter-p11 3 4 10 11pter-11p11 13 14 18 22 Y	1pter-p11.2 3p24.1-p13 3q11.2-q13.31 3q26.1-26.31 3q28-qter 4p15.31-q21.21 5pter-p15.2 9pter-p21.1 9q34.11-qter 11q12.2-q13.3 13q21.3-q21.33 13q33.1-qter 19 22	1pter-p13.1 3 4pter-p16.1 6p22.3-p21.1 9 10 13q12.11-q12.13 16q12.1-q12.2 16q22.3-q24.3 17q12-q21.33 19p13.3-p13.11 19q13.31-qter	2q21.1-qter 3q11.2-q28 5q35.2-qter 7pter-p22.1 8pter-p11.21 11q12.2-q13.3 16pter-p12.1 17pter-p11.2 18q11.2-qter 20q11.21-qter 22	1 3pter-p12.1 9pter-p21 10 19p13.3-p13.2 22q12.2-qter X	3pter-p14.2 9 14	3 9pter-p11 10 14 17pter-p12 19p13.3-p13.2	1pter-p32.3 1p22.3-p21.3 1p21.2-p13.2 2pter-p11.2 2q31.2-qter 6pter-p21.1 9 10q11.23-q24.2 18q11.2-q23 19 21 22	1pter-p33.2 3pter-p11.2 22q12.1-qter	n.d.

n.d.: not detected.

### Fluorescence *In Situ *Hybridization (FISH)

Dual-color interphase FISH analysis was carried out on 4 μm paraffin embedded tissue sections of 42 tumor biopsies. Sections were pretreated using the SPoT-Light Tissue Pre-treatment Kit (Invitrogen, Life Technologies), according to the instructions of the manufacturer. FISH was performed as previously described [22]. Slides were counterstained with 0.1 μM 4,6-diamidino-2-phenylindole (DAPI) in an antifade solution for microscopy.

For analysis of EGFR family members, FISH was performed using the *locus *specific identifier (LSI) EGFR-SpectrumOrange(SO)/CEP7-SpectrumGreen(SG) and PathVysion HER2-SO/CEP17-SG probes (Applied Biosystems/Ambion, Life Technologies, Carlsbad, CA, USA). For evaluation of *PDGFRB/CSF1R *copy numbers and *PDGFRB*/*CSF1R *integrity, the SG-labeled bacterial artificial chromosome (BAC) RP11-21I20 (which maps centromeric to *PDGFRB/*5q33.1 and covers the adjacent *CSF1R *gene) and the SO-labeled RP11-368O19 (which maps telomeric to *CSF1R *and covers the *PDGFRB *gene) DNA probes (both from Research Genetics, Huntsville, AL, USA) were used. In addition, the *PTEN *copy numbers were investigated using dual-color LSI PTEN/CEP10 probe (Applied Biosystems/Ambion).

Hybridization signals were visualized using an epifluorescence microscope (Leica DMRB, Wetzlar, Germany) equipped with a cooled CCD camera and run by the ISIS digital image analysis system (MetaSystems, Altlussheim, Germany). One hundred nuclei were evaluated for the number of red and green signals in different areas corresponding to tumor tissues.

FISH results were classified into five categories according to the percentage of tumor cells with a specific gene/CEP ratio and according to the gene copy number per nucleus: 1) monosomy (1 signal from the gene paralleled by one chromosome centromere signal) or loss (a gene/CEP ratio of <0.6) in >40% of cells; 2) disomy (2 signals from the gene/CEP probes); 3) polysomy (defined as > 2 gene signals per nucleus paralleled by similar increases in chromosome centromeric signals in at least 10% of tumor cells); 4) low level gene amplification (gene/CEP ratio of > 2 in 10%-40% of tumor cells) or 5) high level gene amplification (presence of gene clusters or a gene/CEP ratio of > 2 in ≥40% of analyzed cells).

### Mutation analysis

Mutational analysis was performed on genomic DNA extracted from frozen tumor tissues (n = 13). The sequence coding for the juxtamembrane and/or kinase domains of *PDGFRA *and *PDGFRB *(exons 12, 14 and 18), *KIT *(exons 9, 11 and 17), *CSF1R *(exons 10 to 20) and *EGFR *(exons 18 to 21) genes, were amplified by polymerase chain reaction (PCR), using standard Taq DNA polymerase (Roche Diagnostics, Basel, Switzerland) and the ABI PRISM 9700 (Applied Biosystems). Genomic sequences were obtained from online databases from the National Center for Biotechnology Information (NCBI), and specific primers for amplified fragments were designed using the Primer3 software [23] (http://frodo.wi.mit.edu/cgi-bin/primer3/primer3_www_slow.cgi). Primers sequences are available upon request. The PCR products were purified (QIAquick PCR Purification Kit, QIAGEN, Hilden, Germany) followed by direct bi-directional cycle sequencing using the ABI PRISM 3130 XL Genetic Analyzer (Applied Biosystems, Foster City, CA, USA).

### Western immunoblotting

Cell lysis of frozen tumors (n = 9), SDS-PAGE, and immunoblotting were carried out as previously described [22]. In short, tumor lysate aliquots containing 30 μg of protein were electrophoresed and blotted to PVDF membranes (GE Healthcare, UK). Membranes were blocked in PBS containing 5% blocking reagent (non-fat milk) and immunoblotted sequentially using rabbit antibodies against phospho-EGFR(Tyr1068) (Santa Cruz Biotechnology, Santa Cruz, CA, USA), total EGFR (Santa Cruz Biotechnology), phospho-PDGFRB(Y751) (mc; Cell Signaling, Beverly, MA, USA), total PDGFRB (mc; Cell Signaling), phospho-KIT(Tyr703) (mc; Invitrogen, Life Technologies), total KIT (pc; DAKO), phospho-ERK1/2 (Cell Signaling), total ERK1/2 (Cell Signaling), phospho-AKT (Cell Signaling) and total AKT (Cell Signaling), diluted in 5% blocking reagent. Total β-actin (Sigma Aldrich, St. Louis, MO, USA) was used as a protein-loading and transfer control. The HRP-conjugated anti-rabbit IgG (DAKO) were used at a dilution of 1:2000, and visualized with Enhanced Chemiluminescence (Thermo Scientific, Rockford, IL, USA).

### Receptor tyrosine kinases (RTK) activation profiling using antibody arrays

The activation of RTK and their downstream signaling pathways were analyzed using the Proteome Profiler™ Array kits (ARY001 and ARY003, R&D Systems, Minneapolis, MN, USA) in 12 fresh frozen chordoma tumor specimens. Assays were performed according to the manufacturers' protocol, and using 500 μg of protein lysate per array. The images were captured and the level of RTK activation was visualized with the FUJI mini-LAS3000-plus imaging system (FUJIFILM, Tokyo, Japan) and densitometrically quantified with AIDA software (Raytest isotopenmessgeräte GmbH, Straubenhardt, Germany). The signal intensities of the probes and the local background of the probes were log_2 _transformed in order to obtain a more symmetric distribution, and the difference between these two resulted in a log_2 _transformed ratio (further referred to as log_2_-intensity ratios). For data normalization, within an array and within a membrane the mean log_2_-intensity ratio was calculated and then subtracted from the log_2_-intensity ratio of each probe. Subsequently, the mean of the log_2_-intensity ratios for each kinase within an array was calculated. In the statistical analysis, a linear mixed model was used instead of a one-sample t-test per probe since the arrays or membranes used to measure the probe intensities may differ. The linear mixed model has the log_2_-intensity ratios as responses, the probes as fixed effects and the membrane as random effect per array [24,25]. The alpha level was set at 5%. As multiple testing corrections, the p-values from the tests for the different probes were adjusted to control the false discovery rate as described by Benjamini and Hochberg [26]. The ranking of the probes was based on the adjusted p-values. All analyses were performed with the statistical package SAS (version 9.2), using the procedure PROC MIXED for the linear mixed model.

## Results

### Histopathology and immunohistochemistry

**Figure 1:**
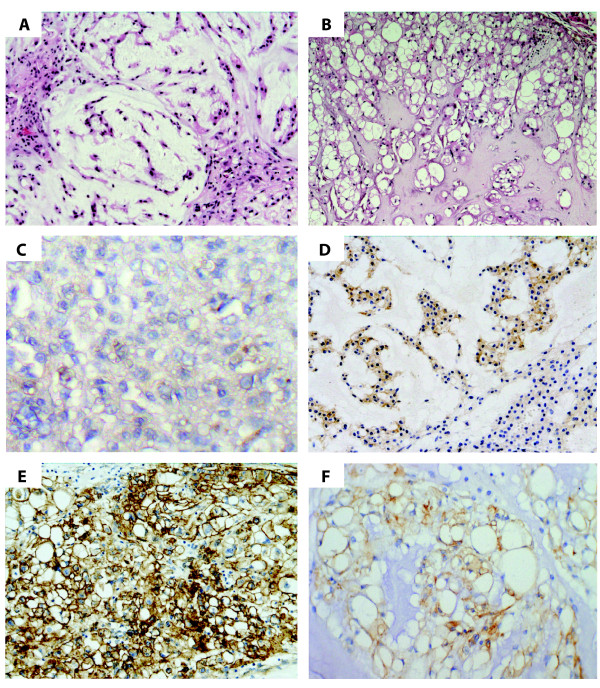
**Histology and EGFR protein expression in chordomas**. A and B/ Examples of histologic appearance of chordomas stained with hematoxylin and eosin (H&E). C - F/ Illustration of chordoma cases with heterogeneous type of positive EGFR immunostaining. F/ The typical physaliphorous cells with abundant vacuolated cytoplasm, showing EGFR membrane staining.

All the chordomas in our cohort were reviewed and classified as conventional chordomas by means of morphology and immunohistochemistry (IHC). They show prominent lobules separated by fibrous septa. The tumor cells are arranged in cords or sheets or may be floating singularly in the abundant myxoid matrix often present. The histologic hallmark is characterized by large tumor cells with abundant vacuolated cytoplasm, referred to as physaliphorous cells [2]. The tumor cells co-express keratin, EMA and S-100 protein. Of the 39 chordomas tested by IHC for EGFR expression, 19 were primary and 20 were advanced lesions. The EGFR immunopositivity was found in 26 out of 39 cases (67%), showing different levels of reactivity (Figure 1, Table 1). Thus, 11 tumors presented with an intense and diffuse cytoplasm membrane positivity in more than 10% of the cells (scored as "3+"), six cases showed intense positive staining but in less than 10% of the cells (scored as "2+"), and nine other cases were considered weakly and discontinuously stained in more than 10% of the cells (scored as "1+"). EGFR expression was more frequently found in advanced tumors compared with primary tumors (80% *versus *58%, respectively). In detail: 15 out of 20 advanced cases stained positive for EGFR *versus *11 out of 19 primary cases. Additionally, when comparing the primary and the advanced stage within patients, in cases 2, 4, 7, 17 and 30: stronger EGFR staining was observed in the advanced in comparison with the primary stage. Case 12 showed intense and diffuse (3+) staining in both the primary and the advanced stage. Case 1 was the only exception, showing stronger EGFR staining in the primary than in the advanced stage. Cases 10, 15 and 31 stained negative for EGFR in the primary stage and stayed negative upon progression. HER2 expression was tested in 16 cases, of which 11 were negative, four displayed low level of staining intensity and one case showed intense positive staining, albeit in less than 10% of the cells. HER2 expression was almost as frequent in primary as in advanced tumors (33% *versus *29%, respectively). The HER2 immunopositivity was associated with EGFR co-expression in all but one lesion, although the level of EGFR expression was heterogeneous.

### aCGH study

**Figure 2:**
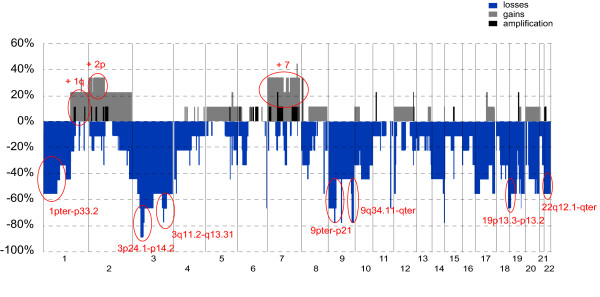
**Frequency (%) of gained and lost regions detected by 4.3K TK aCGH in chordomas**. Gains are shown in grey, losses in blue and amplification in black. Important recurrent gains and losses are circled in red. No rearrangements or high level amplification of genes encoding TK were detected.

Using the whole genome 4.3 K TK-array, we studied copy number aberrations (CNA) in eleven cases for which frozen tissue was available. Ten out of the 11 tumors analyzed showed CNA by aCGH. CNA frequencies were calculated on these ten cases with CNA. Losses were more common than gains, supporting previous findings in chordoma [7]. There was a median of one gain (range 0-5) and seven losses (range 0-14) per tumor. Genomic losses affecting five or more tumors (≥ 50% of cases) were identified on chromosomes 1, 3, 9, 10, 19 and 22 (Table 2 and 3, Figure 2). The smallest common region of chromosome 3 deletion, covering bands 3p24.1-p14.2, was lost in eight cases. Three regions located on the short arm of chromosome 1, i.e. 1pter-p33.2, 1p22.3-p21.3 and 1p21.2-p13.2, were recurrently lost in six, five and five cases, respectively. Whole chromosome 9 loss was observed in four cases, and the region 9q34.11-qter, involving among others the *TSC1 *tumor suppressor gene, was lost in one additional case. Furthermore, the region 9pter-p21 was lost in three extra cases of our cohort. Of note, homozygous deletion of the chromosomal sub-band 9p21.3 (the region containing the *CDKN2A *tumor suppressor gene) was found in three of analyzes tumors. The entire chromosome 10 was lost in four cases and the region 10q11.23-q24.2, encompassing the tumor suppressor *PTEN*, was lost in another case. Losses that implicated chromosome 19, with the commonly deleted region 19p13.3-p13.2, were found in five cases. Total or partial chromosome 22 deletions, with the common region 22q12.2-qter, were recorded in six chordomas. The most common gain was the gain of the entire chromosome 7, observed in three chordoma cases (Table 2). Notably, the genes coding for the EGFR, MET, LMTK2, EPHA1, EPHB4 and EPHB6 proteins are mapped on chromosome 7. No amplifications or rearrangements within the 90 known TK were detected in our cohort of chordomas.

**Table 3:** Recurrent copy number losses in chordoma cases by aCGH

Regions lost in ≥ five cases			
**Chordoma cases (#)**	**Cytogenetic location**	**Frequency**	**Candidate genes**

4b, 5, 7b, 10b, 15a, 16	1pter-p33.2	0.60	*RUNX3*
4b, 5, 7b, 10b, 15a	1p22.3-p21.3	0.50	
3, 4b, 5, 7b, 10b, 13, 14, 16	3p24.1-p14.2	0.80	*RBM5, FHIT, PTPRG*
4b, 5, 7b, 9, 14	3q11.2-q13.31	0.50	
4b, 5, 7b, 9, 14	3q26.1-26.31	0.50	
3, 5, 7b,10b, 13, 14, 15a	9pter-p21	0.70	*CDKN2A*
3, 5, 7b, 13, 15a	9q34.11-qter	0.50	*TSC1*
4b, 7b, 10b, 14, 15a	10q11.23-q24.2	0.50	*PTEN*
5, 7b, 10b, 14, 15a	19p13.3-p13.2	0.50	
4b, 5, 9, 10b, 15a, 16	22q12.2-qter	0.60	*CHEK2*

### FISH analysis

**Figure 3:**
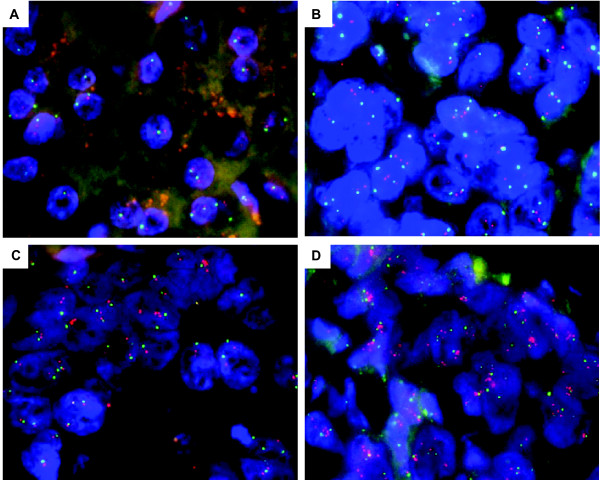
**Representative examples of dual-color interphase FISH images on paraffin sections in chordomas**. Detected by the co-hybridization of SpectrumOrange labeled *EGFR *DNA probe (red signals) and SpectrumGreen labeled chromosome 7 CEP probe (green signals). (A) Case 1a, showing *EGFR *disomy. (B) Case 10a reveals *EGFR *polysomy. (C) Case 20 shows low level amplification in < 10% of nuclei. Of note, this amplification is not detected by aCGH. (D) Case 12b, showing high level amplification of *EGFR *in > 40% of nuclei.

The gene copy numbers of the *EGFR, HER2, CSF1R*/*PDGFRB *and *PTEN *were analyzed by FISH (Figure 3, Table 1). Sixteen out of 42 tumors analyzed revealed disomy for *EGFR*, while 16 (38%) cases displayed polysomic cell clones. Two cases showed chromosome 7 polysomy. Only a small fraction of tumors (four cases) presented with *EGFR *amplification, and only in two cases at high level. Notably, four cases showed *EGFR *loss. The gene copy number of *HER2 *was also analyzed in all cases, and six specimens revealed polysomy of *HER2*. Three cases showed *HER2 *loss. Of note, half of the *HER2 *gains were not detectable by aCGH, probably due to a low number of neoplastic cells in these specimens. Copy number gains of both, *EGFR *and *HER2 *genes, correlated well with HER2 immuno-positivity by IHC. Of the 34 cases analyzed, 13 tumors were polysomic for *CSF1R*/*PDGFRB *and two revealed loss of *CSF1R*/*PDGFRB*; the remaining presented disomy for these genes. The tumor suppressor *PTEN *was lost in seven out of 18 analyzed tumors.

### Mutation analysis

No activating mutations of *EGFR, CSF1R, PDGFRB, PDGFRA *or *KIT *in examined genes' exons were found in any of the 13 analyzed cases (Table 1).

### RTK phosphorylation profiling using phospho-RTK and phospho-kinase antibody arrays

**Figure 4:**
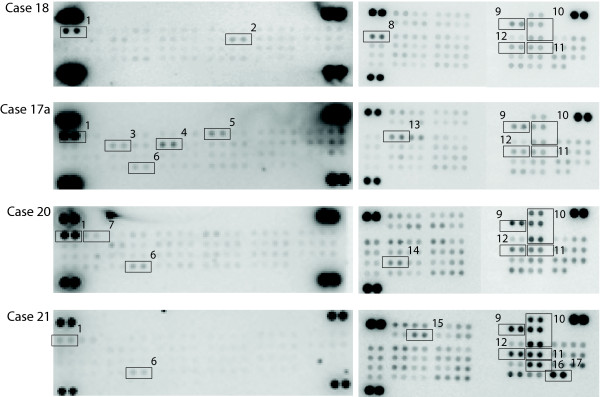
**Representative images from phospho-RTK (left panel) and phospho-kinase (right panel) arrays from chordoma cases 18, 17b, 20 and 21**. The EGFR and EPHB2 TK are frequently activated and downstream RTK signaling intermediates are activated consistently in chordomas. Each kinase is spotted in duplicate. The pairs of dots in each corner are positive controls. Each pair of the most positive kinase dots is denoted by a numeral, with the identity of the corresponding kinases listed as follows: 1) EGFR, 2) CSF1R, 3) MSPR, 4) PDGFRB, 5) FGFR3, 6) EPHB2, 7) HER2, 8) TOR, 9) AKT, 10) TP53, 11) RSK1/2/3, 12) S6K, 13) CREB, 14) YES, 15) MSK1/2, 16) RSK1/2, 17) eNOS.

The results of the RTK- and kinase-analysis of 12 and 10 chordoma samples respectively are shown in Table 4 and examples are depicted in Figure 4. The probes are ranked according to their false discovery rate (fdr) adjusted p-value. The column "Estimate" shows the estimate mean log_2_-intensity ratio for each RTK or kinase over all experiments. The first three RTK-probes and the first twelve kinase-probes in Table 4 have a log_2_-intensity ratio significantly larger than zero at the alpha level of 5%. Thus, the EPHB2, EGFR and macrophage-stimulating protein receptor (MSPR) were found to be significantly activated in chordoma. Although present in some of the analyzed specimens, activation of the PDGFRB, FGFR3, CSF1R and ERBB4 was not statistically significant in our study. Strikingly, there was no detectable activation of KIT or VEGF receptors. By analyzing the signaling pathways (the profiles of 46 kinases and protein substrates), AKT, RSK1/2/3, TP53, MSK1/2, YES, p38a, p70 S6K, CREB and SRC were the most frequently and strongest phosphorylated proteins in our cohort. Interestingly, SRC family members, as SRC and YES, were recurrently activated in chordoma. Furthermore, kinase-array revealed the activation of downstream effectors of both, the PI3K/AKT/mTOR and RAS/RAF/MAPK pathways.

**Table 4:** Significantly phosphorylated RTK and kinase sites in chordoma using Proteome Profiler arrays, ranked based on p-value

Probe name	Estimate	Standard Error	t-value	Raw p-value	fdr adjusted p-value
Phospho-RTK					

**EPHB2**	**0.1285**	**0.0263**	**4.9**	**6.6931E-07**	**2.8111E-05**
**EGFR**	**0.6762**	**0.1694**	**3.99**	**3.8547E-05**	**0.0008**
**MSPR**	**0.1241**	**0.0426**	**2.91**	**0.0019**	**0.0266**
PDGFRB	0.0848	0.0334	2.54	0.0057	0.0600
FGFR3	0.1022	0.0484	2.11	0.0177	0.1487
CSF1R	0.0887	0.0445	1.99	0.0236	0.1652
ERBB4	0.0160	0.0289	1.78	0.0379	0.2272

Phosphorylated kinase site					

**AKT (T308)**	**0.3117**	**0.0313**	**9.95**	**3.1253E-21**	**1.5001E-19**
**RSK 1/2/3 (S380)**	**0.1747**	**0.0212**	**8.25**	**1.2388E-15**	**2.9731E-14**
**TP53 (S46)**	**0.2394**	**0.0336**	**7.14**	**2.3075E-12**	**3.6920E-11**
**MSK 1/2 (S376/S360)**	**0.1557**	**0.0256**	**6.09**	**1.3564E-09**	**1.6277E-08**
**YES (Y426)**	**0.1639**	**0.0288**	**5.69**	**1.2512E-08**	**1.2012E-07**
**TP53 (S15)**	**0.2533**	**0.0469**	**5.41**	**5.5176E-08**	**4.4141E-07**
**p38a (T180/Y182)**	**0.2858**	**0.0625**	**4.57**	**3.2798E-06**	**2.2490E-05**
**p70 S6K (T421/S424)**	**0.1086**	**0.0242**	**4.49**	**4.6993E-06**	**2.8196E-05**
**CREB (S133)**	**0.3273**	**0.1018**	**3.21**	**0.0007**	**0.0038**
**RSK 1/2 (S221)**	**0.0707**	**0.0246**	**2.87**	**0.0022**	**0.0104**
**SRC (Y419)**	**0.0934**	**0.0349**	**2.68**	**0.0038**	**0.0158**
**TP53 (S392)**	**0.1237**	**0.0464**	**2.67**	**0.004**	**0.0158**
TOR (S2448)	0.2407	0.1258	1.91	0.0284	0.105
JUN (S63)	0.0863	0.0533	1.62	0.053	0.1818
HSP27 (S78/S82)	0.1048	0.0691	1.52	0.0647	0.2016
eNOS (S1177)	0.2002	0.1331	1.50	0.0672	0.2016
STAT1 (Y701)	0.0465	0.0318	1.46	0.0725	0.2048
STAT5b (Y699)	0.0380	0.0286	1.33	0.0921	0.2457
LYN (Y397)	0.0351	0.0283	1.24	0.1079	0.2725
STAT6 (Y641)	0.0309	0.0284	1.09	0.1382	0.3317
STAT5A (Y699)	0.0656	0.0715	0.92	0.1791	0.4093
FYN (Y420)	0.0587	0.0768	0.76	0.2239	0.4884
STAT5A/B (Y699)	0.0168	0.0366	0.46	0.3229	0.6739
ERK1/2 (T202/Y204. T185/Y187)	0.0284	0.0708	0.40	0.3447	0.6894

* The probes written in bold have a log2-intensity ratio significantly larger than zero at the α-level of 5%.

### Western immunoblotting

**Figure 5:**
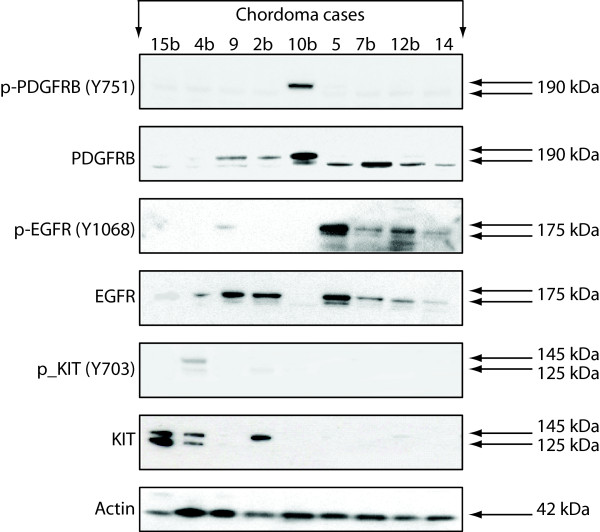
**Western immunoblot of nine chordoma cases**. The immunoblot confirms the frequent expression of EGFR and PDGFRB, and frequent activation of EGFR, but not of PDGFRB and KIT proteins. Equal amounts of total protein extracts from nine tumors were separated on a gel, immunoblotted and then probed with the indicated antibodies.

The consistent protein expression of EGFR and PDGFRB and the recurrent activation of EGFR were confirmed by Western blotting (Figure 5). The expression status of EGFR in all cases was in agreement with the results obtained by IHC (Table 1 Figure 5). Briefly, cases 15b and 10b showing only faint EGFR staining on the Western blot were scored negative by immunostaining. All other cases, presenting clear or intense EGFR expression by Western, were immune-scored accordingly as "1+", "2+" or "3+". Two specimens were analyzed in parallel by Western immunoblotting and RTK antibody array. The strong EGFR activation of case 12b detected by Western was confirmed by RTK antibody array. In case 4b, EGFR was expressed but not activated by Western. However, intermediate activation of EGFR was disclosed for this lesion by RTK antibody array. This apparent difference could be ascribed to the fact that the antibody used for Western blot detects the phosphorylation status of just one EGFR tyrosine residue (Y1068), while the antibody array detects the phosphorylation of all tyrosine residues on the EGFR protein. Furthermore, different pieces of the tumor were used as starting material for both experiments, which may bring about differences, as chordomas are proven to be heterogeneous lesions. By Western immunoblot, PDGFRB was found to be expressed in all chordomas analyzed, although only one case (#10b) also presented activated PDGFRB. KIT protein expression and low level activation was found in three and two cases respectively.

## Discussion

Recent reports suggest that RTK might be essential for the survival or proliferation of chordoma tumor cells. Therefore, targeting RTK may offer new therapeutic options for chordoma treatment. Nevertheless, there are important discrepancies between the reported results, which are most likely due to differences in the relative sensitivities of the methods used or heterogeneity of the material analyzed. Moreover, the characterization of chordoma in most studies is rarely based on parallel multiple techniques. Our objective was to characterize this rare entity in search for relevant therapeutic targets using a wide range of methodological approaches.

Whole genome 4.3 K TK-array CGH revealed moderately complex CNA across the genome in all but one examined cases, with losses more common than gains. The CNA found in our cohort were in accordance with previously recognized imbalances in chordomas [3,4,7,27-29]. No deletions or gains common to all samples were found, confirming that chordomas are genetically heterogeneous tumors.

Importantly, we did not identify any amplifications or rearrangements involving genes coding for TK. Interestingly though, the most recurrent copy number gain, found in three out of ten cases, involved the entire chromosome 7. Gain of chromosome 7 is frequently reported in chordomas, and multiple genes that encode TK are located on chromosome 7, including the *EGFR *[3,4,7,27-29]. Accordingly, copy number gains involving the *EGFR *locus, were found by FISH in 22/42 (52%) of our cases. Polysomy of the *EGFR*/*ERBB1 *gene was previously reported in a subset of chordomas, and the EGFR is an interesting target for therapy in chordoma based on the availability of targeted molecular inhibitors [8,16]. Additionally, the status of the gene encoding HER2, a close family member and important dimerization partner of EGFR, was investigated. Copy number gains of *HER2 *were identified in 6/42 (14%) of cases. Noteworthy, copy number gains of *HER2 *were exclusively found in recurrent or metastatic cases in our cohort, further suggesting its possible association with poor clinical outcome.

Losses of large chromosomal regions are typically found in chordoma. In this study, losses revealed by aCGH predominantly involved chromosome 3; the smallest overlapping region of deletion, 3p24.1-p14.2, was lost in eight out of ten analyzed cases. This region contains multiple genes, including *RBM5, FHIT *and *PTPRG*, but their involvement in chordoma pathogenesis has yet to be determined. Loss of the 9pter-p21 region, another frequent feature revealed by aCGH analysis, was found in seven out of ten tumors. Importantly, in three cases the region was homozygous lost. The losses encompassed the tumor suppressor genes *CDKN2A *and *CDKN2B*, which are frequently deleted in many tumor types [30,31]. Correspondingly, Hallor and co-workers observed loss of the *CDKN2A *locus with an incidence of 70% in chordoma, and with an even higher frequency considering just metastasizing lesions [7]. Accordingly, loss of expression of the CDKN2A protein in chordoma was also previously shown by immunostaining [32]. Other recurrent losses, observed in the present study by aCGH, involved regions carrying the tumor suppressors *PTEN*/10q23.31* , CHEK2/*22q12.1 and the transcription factor *RUNX3*/1p36.11, all previously described in chordomas [7].

In order to characterize the compendium of co-activated RTK in chordoma, we used an antibody array that allows the simultaneous characterization of the phosphorylation status of 42 different RTK. Most importantly, the EGFR kinase was consistently activated in all 12 investigated cases. Furthermore, statistical analysis showed that EGFR activation was significant for chordomas, based on the analysis of our cohort. The activation of EGFR in chordoma was previously shown by other groups, although the reported frequencies of the EGFR activation in chordoma vary significantly [8,16]. By RTK antibody array Tamborini and co-workers reported EGFR, HER2 and HER4 activation in 6/7 (86%), 5/7 (71%) and 3/7 (43%) of cases, respectively [8]. However, using immunoprecipitation assay, EGFR and HER2 were phosphorylated in respectively 17/22 (77%) and 6/14 (43%) of their cases [8]. Using the same RTK antibody array, Shalaby and colleagues recently showed activation of HER2, MSPR, EPHB2 and MER for the U-CH1 chordoma cell line and the three tested chordoma cases [16]. In our study, we found significant activation of EGFR, HER2 and HER4 in respectively 12, one and one out of 12 cases, using the same antibody arrays. Interestingly, the frequent activation of PDGFRB in chordomas [21/22 (95%) of cases] was described in the study by Tamborini and collaborators [8]. In contrast, we found activation of PDGFRB only in five out of 12 (42%) chordomas, using the same antibody RTK arrays and using the value of the mean plus the standard deviation within an array as the cut-off. However as indicated by statistical analysis, PDGFRB activation was not significant in our cohort. This discrepancy might be attributable to the heterogeneity of chordoma tumors, the quality of the frozen tumor tissue used for the analysis, modifications of the technique and/or to subsequent dissimilar analysis of the data. Thus, Tamborini and co-workers used high-concentrated (e.g. 2 mg/array) protein lysate per array in their study [8]. In contrast, we performed the experiments according to the manufacturers' recommendations which indicate 500 μg of total protein as the maximum amount to be used for each array. In addition, we have performed an extensive statistical analysis of the data by using a linear mixed model. Our statistical analysis included a multiple testing correction. The linear mixed model avoids the use of an arbitrarily chosen cut-off that can lead to overestimation of the activation of RTK and to uncertainty about the results. Notably, statistical analysis was never described by others in reports published so far in reference to RTK proteome profiling kits, thus the statistical significance of reported data is unknown. Importantly, we also found two other RTK: EPHB2 and MSPR, to be significantly activated in chordoma. The activation of EPHB2 was recently described in one chordoma study [16]. The role of EPHB2 in chordoma development and progression needs to be further evaluated. In general, EPHB2 function depends on the tumor type and signaling context of the neoplastic cell. The EPHB2 has a tumor suppressive role in colon carcinoma; in contrast, EPHB2 promotes cell proliferation in adenomas and normal intestinal epithelium. Notably, it was recently shown in mice models that the intrinsic kinase activity of EPHB2 conveys mitogenic signals [33]. It is of interest that imatinib mesylate is as an inhibitor of EPHB mitogenic signaling. The MSPR/RON tyrosine kinase is a member of the MET family of RTK. MET expression was shown previously in chordomas by several other groups, but MSPR expression and activation was only recently reported in all three investigated chordomas by Shalaby and co-workers [16]. As it is the case with its better-known family member, MET, several lines of evidence suggest a role for RON in human cancer. Generally, RON overexpression is associated with poor clinical outcome and metastasis [34]. Foretinib, an oral multi-kinase inhibitor of MET, RON, AXL and VEGFR, is currently in phase I and II clinical testing [35].

The multiple RTK co-activation is not a distinctive feature of chordomas, because similar patterns were reported in other tumor types, such as colon adenocarcinomas, intimal sarcomas, glioblastomas or osteosarcomas [36-38]. Importantly, the simultaneous activation of multiple RTK provides the tumor cells with reduced dependence on a single RTK for the maintenance of critical downstream signaling, and thus renders such tumors refractory to single-agent RTK inhibition.

The conflicting results on the frequency of EGFR, HER2, PDGFRB expression and activation, and also copy number alterations in chordoma, might be due to differences in sensitivity of the techniques used. In addition, even if using the same technique, there are important variations in methodology between different laboratories, with many confounding factors contributing to the inconsistencies, e.g. the different type and source of the antibodies used in the immunohistochemical studies. When immunostaining is considered, it is well known that the way of tissue fixation influences outcome [39]. Tumor specimens are frequently retrieved from archives, and in case they are not preserved well, this may give rise to false negative cases. The lack of sensitivity of IHC to identify low expression levels of EGFR was comprehensively illustrated in colorectal cancer [40]. Similarly, chordoma immunostaining might also show inconsistencies associated with these methodological problems. Along this line, Weinberger and co-workers found EGFR and HER2 expression in respectively 12 (100%) and seven (58%) out of 12 chordomas, using IHC on tissue micro-arrays (TMA), while Shalaby and colleagues showed EGFR expression and activation in respectively 69% (79/114) and 50% (56/115) of chordoma cases by the same technique, and while Fasig and co-authors reported EGFR activation in nine out of 21 (43%) cases [12,15,16]. By conventional immunostaining, we have also found that EGFR and HER2 are expressed in chordomas, albeit in a lower fraction of cases 26/39 (67%) and 5/16 (31%), respectively. In contrast to Weinberger and co-workers, however, we found more frequent EGFR expression in advanced (15/20, 75%) rather than in primary (11/19, 58%) lesions. Again in contrast to Weinberger and co-workers, we did find a positive correlation between HER2 expression and EGFR expression, which is in line with the HER2/EGFR heterodimers formation in chordomas reported by other groups [8,12]. Moreover, we did not find a significant correlation between *EGFR *and *HER2 *gene status and their expression by immunostaining, this phenomenon was also described in colorectal cancer [40,41].

The circuitry of intracellular signalling downstream of RTK is an area of dynamic investigations in many cancer types and advances in the characterization of this signalling allows better selection of appropriate therapeutic agents. In the present study, we analyzed the activation of important effectors of signalling downstream of RTK. Using kinase antibody arrays, AKT was the most frequent (found in nine out of ten cases analyzed) and highest phosphorylated in chordomas. Similarly, Presneau and co-workers found AKT activation in 45 out of 49 (92%) chordomas analysed by TMA, and Tamborini and colleagues in 21 out of 22 chordomas (95%) using Western blotting [8,42]. The AKT protein transduces signals to several effector molecules, including TSC1/2. More specifically, AKT inhibits TSC1/2 and hereby relieves inhibition of mammalian target of rapamycin (mTOR), which functions downstream of TSC1/2. This occurs in part by phosphorylating two substrates, p70S6 kinase (S6K) and eukaryotic initiation factor 4E-binding protein 1 (4E-BP1). Of note, p70S6K was activated in five and mTOR in three of our ten chordoma cases analyzed by kinase antibody arrays. These data are in accordance with previously published data [8,15,43]. The phenomenon that p70S6K was activated in p-mTOR negative chordomas was found in multiple studies [8,42]. The discrepancy in the prevalence of the activated proteins between the reported results is most likely due to differences in the relative sensitivity and specificity of the methods. This is well illustrated in a study by Dobashi and co-workers, who found activated mTOR in all five cases using immunohistochemistry, but only in one case using Western immunoblotting [44]. Nevertheless, the involvement of the AKT/mTOR pathway in chordoma is clear. Importantly, efficient inhibition of the human chordoma cell line UCH-1 by PI-103, a dual PI3K and mTOR inhibitor, was recently reported [43]. Notably, it was recently shown that AKT activation persists in the UCH-1 chordoma cell line following treatment with the EGFR inhibitor tyrphostin [16].

Furthermore, by kinase antibody arrays, we also found effectors of RAS/ERK1/2 signaling to be significantly activated in chordoma, like ribosomal S6 kinases (RSK) 1/2/3, the CREB transcription factor and the chromatin associated kinase p38. More downstream are the mitogen- and stress-activated protein kinases, MSK1 and the closely related isoform MSK2. These are nuclear kinases that are activated by the ERK1/2 and p38 MAPK signaling cascades [45]. Additionally, the SRC family members, SRC and YES, were also activated. These pathways were not extensively analyzed in chordoma by other groups, except for ERK1/2, which was described to be consistently strongly phosphorylated in chordoma by Tamborini and co-workers [8]. Nevertheless, these activated proteins are all confounding factors that might offer the tumors redundancy, making them less responsive to upstream RTK and AKT pathway inhibition.

Oncogenes often cooperate with additional mutations that disrupt tumor suppressor pathways. Phosphatase and tensin homologue deleted on chromosome ten (PTEN), is an important negative regulator of the AKT/mTOR pathway, which when not expressed contributes to constitutive phosphorylation of AKT and activation of downstream effectors. *PTEN *loss is also frequently found in chordomas. We observed loss of *PTEN *in five out of ten cases by aCGH, and in seven out of 18 (39%) cases by FISH. Presneau and co-workers recently revealed loss of PTEN protein expression in seven out of 43 (16%) cases by IHC and semi-quantitative RT-PCR [42]. Han and co-workers showed negative PTEN staining by IHC in six out of ten sporadic chordoma [46]. Just like in our cases, they did not find any correlation between loss of PTEN and advanced disease. TSC1 is another critical tumor suppressor, implicated downstream in the PI3K/AKT and RAS/ERK pathways. In particular, upon growth factor activation, AKT, ERK and p90 ribosomal S6 kinase 1 (RSK1) participate in TSC protein complex inhibition, hereby critically regulating cell growth and proliferation. Chordomas are reported in patients with tuberous sclerosis complex (TSC), an autosomal dominant disorder typified by hamartomas in several organs, epilepsy, mental retardation and behavioural problems. TSC is caused by germline mutations in the *TSC1 *or *TSC2 *genes and the loss of the corresponding wild type allele. The chromosomal region 9q34.13, where the *TSC1 *gene is localized, is also frequently lost in sporadic chordomas [7]. By aCGH, we found loss of the region 9q34.11-qter, encompassing the gene coding for TSC1, in five out of ten cases. Hallor and co-workers showed loss of this region in about 25% of 21 cases analyzed by aCGH. In contrast, Presneau and co-workers found disomy for *TSC1/2 *by FISH in all of their 28 cases [42]. Generally, the consistent activation of AKT, the frequent activation of p70S6K and of mTOR, together with frequent loss of the *TSC1 *and *PTEN *genes, all suggest an important role for the PI3K/AKT pathway in chordoma.

## Conclusions

In summary, we found that EGFR is the strongest and most frequently activated RTK in chordomas, and therefore becomes a possible target for therapy. Lack of significant *EGFR *amplification and *EGFR *mutations suggests activation by autocrine/paracrine ligand stimulation. PDGFRB is also activated in chordomas, but with a lower frequency and/or to a lower level, which might not be detectable by some current standard techniques. In the light of these findings, chordoma patients may benefit from treatment with multi-kinase inhibitors targeting both EGFR and PDGFR. Furthermore, many other RTK are activated in subsets of chordomas; these are likely to increase treatment resistance in these tumors. These results are currently only hypothesis-generating, and additional *in vitro *studies addressing the impact of inhibitors of RTK and their downstream effectors on chordoma tumor cells would be extremely useful in determining the dominant and alternative RTKs in these tumors. As chordomas are bone tumors, with a rigid, mineralized extracellular matrix, *ex-vivo *studies on primary neoplastic chordoma cells will be difficult. Recent advances in computational biology and network-based technologies generating predictive models might be more of use [47].

In conclusion, the consistent activation of AKT, the recurrent activation of upstream EGFR and of downstream effectors like p70S6K and mTOR, together with frequent loss of *TSC1 *and *PTEN *gene loci, all indicate that the PI3K/AKT pathway is an important mediator of transformation in chordoma. Targeting this pathway is likely to yield attractive data that will enlighten the design of appropriate therapies. Individualized therapeutic approaches depending on the genetic context of a particular tumor are likely to be the most successful.

## List of abbreviations

4EBP1: eukaryotic translation initiation factor E4-binding protein 1; BAC: bacterial artificial chromosome; CNA: copy number alterations; CSF1R: colony-stimulating factor 1 receptor; DAPI: 4.6-diamidino-2-phenylindole; EGFR: epidermal growth factor receptor; ERK1/2: extracellular signal-regulated kinase; fdr: false discovery rate; HER2: v-ERBB2 Avian erythroblastic leukemia viral oncogene homolog 2; IHC: immunohistochemistry; IS: intimal sarcoma; MEK: mitogen-activated kinase kinase kinase 1; mTOR: mammalian target of rapamycin; NCBI: National Center for Biotechnology Information; PCR: polymerase chain reaction; PDGFR: platelet derived growth factor receptor; PI3K: phosphatidyl inositol 3 kinase; PKB or AKT: protein kinase B; RTK: receptor tyrosine kinase; S6K: ribosomal protein S6 kinase; SG: spectrum green; SO: spectrum orange; TK: tyrosine kinase; TKI: tyrosine kinase inhibitors; TMA: tissue microarrays.

## Competing interests

The authors declare that they have no competing interests.

## Authors' contributions

BD carried out the mutation analysis, participated in the aCGH data evaluation, Western immunoblotting analysis and antibody array analysis, and drafted the manuscript. FM carried out the histopathological experiments and analysis and participated in the draft of the manuscript. GF participated in the antibody array experiments and analysis and histopathological analysis. MA performed the statistical analysis of the antibody arrays. VV carried out the FISH, aCGH, Western immunoblotting and antibody array experiments. AW performed the aCGH analysis and participated in the antibody array analysis. MDR participated in the design and coordination of the study and helped to draft the manuscript. RS contributed tumor samples for this study, participated in the design of the study and critically revised the manuscript. All authors read and approved the final manuscript.
